# Principles of Best Diagnostic Practice in Tissue Repair and Wound Healing: An Expert Consensus

**DOI:** 10.3390/diagnostics11010050

**Published:** 2020-12-31

**Authors:** David G. Armstrong, Karen Bauer, Greg Bohn, Marissa Carter, Robert Snyder, Thomas E. Serena

**Affiliations:** 1 Keck School of Medicine of USC, Southwestern Academic Limb Salvage Alliance (SALSA), Los Angeles, CA 99033, USA; armstrong@usa.net; 2 Department of Surgery, University of Toledo Physicians, Toledo, OH 43614, USA; karen.bauer@utoledo.edu; 3 General Surgery Tawas St Joseph Hospital & Ascension St Joseph Hospital, Tawas City, MI 48763, USA; gregbohn2@aol.com; 4 Owner of Strategic Solutions, Bozeman, MT 59718, USA; mcarter@strategic-solutions-inc.com; 5 Clinical Research Barry University SPM, Brand Research Center, Barry University, Miami, FL 33321, USA; drwound@aol.com; 6 SerenaGroup^®^ Inc., Cambridge, MA 02140, USA

**Keywords:** chronic wound, host proteases, bacterial proteases, virulence factors, wound diagnostics

## Abstract

Chronic wound treatment currently relies heavily on visual assessment by clinicians; however, the clinical signs and symptoms of infection and inflammation are unreliable in chronic wounds. The specialty of wound care has witnessed the advent of advanced interventions, such as cellular and/or tissue based products (CTP). The success of advanced therapies relies on preparing the wound bed by reducing bacterial burden and inflammation. The lack of diagnostics in chronic wound care leads to uncertainty in the adequacy of wound bed preparation. Recent research suggests that two novel point-of-care diagnostic tests can assist in the detection of chronic inflammation known as elevated neutrophil derived protease activity (EPA) and bacterial pathogenesis known as bacterial protease activity(BPA) in chronic wounds. Despite the evidence, however, clinicians report that incorporating diagnostics into every day practice is challenging and across the globe, they have requested guidance on their use. Methods and Recommendations: A panel of wound care experts, experienced with these tests, met to develop guidelines on their use in wound care practice. The consensus panel concluded that the clinician should test for BPA first. The panel maintained that the risk of invasive infection resulting from the presence of pathogenic bacteria was the greatest threat to the patient’s health. If the BPA test is negative, the panel recommended testing for EPA. In addition, it was suggested that if the wound failed to progress after the elevated BPA was treated and subsequent testing was negative for BPA, the clinician should consider testing for EPA. Conclusions: In this manuscript, the consensus panel suggests pathways for testing, treating, and retesting for EPA and BPA. The panel expects that following the algorithm has the potential to improve healing outcomes, result in more cost-effective use of advanced therapies, and improve antimicrobial stewardship by guiding antimicrobial use.

## 1. Introduction

Medicare estimates that over 8 million Americans suffer from chronic wounds at a cost ranging from 18.1 to 96.8 billion dollars [1]. The increased incidence of nonhealing wounds coincides with the aging of the population and the rising incidence of diabetes and obesity [2]. Despite the introduction of advanced wound dressings, negative pressure wound therapy, cellular and/or tissue based products (CTP) and oxygen therapies, less than half of wounds heal after 12 weeks of treatment [3]. The key to wound healing and the success of advanced therapies is adequate wound bed preparation consisting of debridement, proper moisture balance, reduction in bacterial burden and inflammation, offloading for diabetic and pressure ulcers, and compression for venous leg ulcers [4]. In current practice, however, deciding when a wound bed is adequately prepared is problematic, if not impossible.

The specialty of wound care developed without the benefit of diagnostic testing for inflammation or bacterial burden [5,6,7,8]. Today, clinicians rely on clinical signs and symptoms (CSS) to diagnose excessive inflammation and elevated bacterial levels in non-healing wounds; however, CSS are unreliable [9]. In a large multicenter clinical trial, the average sensitivity of CSS in detecting bacteria was only 15% [10]. Recent evidence suggests that two novel point-of-care diagnostic tests may fill the unmet need for wound diagnostics [11,12].

The availability of point-of-care diagnostics to wound practitioners across the globe has generated questions on guidelines for their use. In this manuscript a consensus panel of wound care experts addresses the most commonly asked questions: Which test should be used first (EPA or BPA), what are the best therapies for positive tests, and when is the best time to retest?

## 2. The Technology

### 2.1. Excessive Inflammatory Protease Activity (EPA)

The EPA test (Woundchek™ laboratories, Gargrave, UK) provides a qualitative assessment of human inflammatory protease activity (EPA) in the wound. Specifically, a positive EPA indicates elevated levels of matrix-metalloproteases (MMPs) 2, 8 and 9 and human neutrophil-derived elastase. A multicenter clinical trial evaluating the point-of-care test demonstrated that 90% of wounds with EPA failed to progress toward healing (median HNE 2.6 mU/110 μL (range 0–108) and median total MMP 12.6 U/110 μL (range 0–476)) [11]. In addition, several studies have found elevated inflammatory human (host) protease activity associated with non-healing chronic wounds: a weighted average of eight studies covering 503 patients demonstrated EPA in 22% of non-healing chronic wounds [13]. Conversely, low protease activity is found in wounds on a healing trajectory or in chronic wounds in which the etiology of delayed wound healing is not secondary to excessive MMP and HNE activity.

In addition, studies have shown that wound bed preparation that does not reduce protease levels is associated with skin graft failure. In contrast, grafts with low protease activity prior to grafting had 85–100% successful graft take [14,15].

### 2.2. Bacterial Protease Activity (BPA)

**Figure 1:**
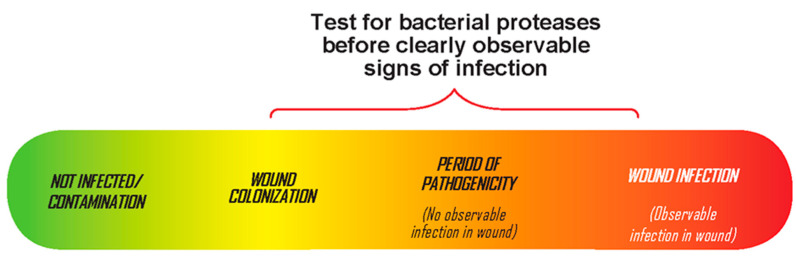
The continuum of bacteria in chronic wounds. Testing for pathogenic bacteria prior to observable signs of infection.

The BPA test (Woundchek™ laboratories, Gargrave, UK) provides a qualitative assessment of bacterial protease activity from the most common bacteria in chronic wounds (*Staphylococcus aureus*, *Pseudomonas aeruginosa*, *Proteus mirabilis*, and *Enterococcus faecalis*). It detects elevated bacterial proteases called virulence factors that correlate with bacterial pathogenicity. In a multicenter clinical trial, elevated BPA was associated with delayed wound healing. In addition, the detection of increased BPA permitted the identification of pathogenic bacteria in the wound prior to the onset of clinical signs and symptoms [12]. Ideally, clinicians would identify and treat pathogenic bacteria in the wound prior to the onset of CSS (Figure 1).

## 3. Methods

An interactive group technique (IGT) was employed to discuss the incorporation of wound diagnostics into clinical practice. A panel of clinician-scientists and trialists chosen for their contributions to the field of wound healing, reviewed the evidence for two point-of-care diagnostics (elevated protease activity and bacterial protease activity, WoundChek™ Laboratories. Gargrave, UK) prior to convening on 16 July 2020. The first task for the group was to identify the most commonly asked questions by clinicians concerning the diagnostic tests. The panel choose to address three questions: (1) which diagnostic test should be used first?; (2) what are the best therapies for positive tests?; and (3) when is the best time to retest?

In formulating recommendations, the panelists considered the science behind excessive inflammatory and bacterial proteases including their role in the delayed healing of chronic wounds. They also focused on the importance of wound bed preparation in promoting wound healing and increasing the effectiveness of advanced therapies. In addition, the evidence for interventions to treat excess inflammation and increased bacterial burden was reviewed.

The panel members’ consensus followed the World Union of Wound Healing Societies’ principles of best diagnostic practice [16]. Briefly, the document states that the process of diagnosis identifies a disease or medical condition from the patient’s signs and symptoms and from any tests performed. In the effective treatment of patients with wounds, the diagnostic process will:

- determine the cause of the wound;
- identify any comorbidities/complications that may contribute to the wound or delay healing;
- assess the status of the wound;
- help to develop the management plan.

**Figure 2:**
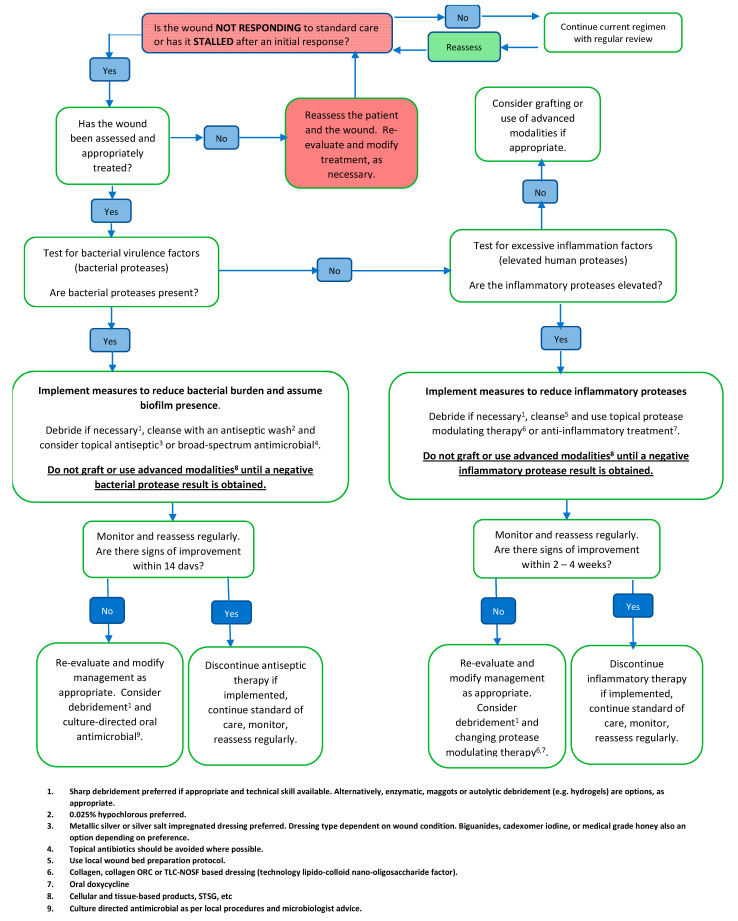
Suggested pathway for the use of point of care wound diagnostic tests [17,18].

Once a management plan has been implemented, repetition of elements of the diagnostic and assessment process (e.g., re-examination and repetition of tests) can assist in monitoring the healing progress and identifying complications such as invasive infection. Re-evaluation may also indicate the need for different tests and/or for adjustment of the management plan. The panel developed a pathway for the use of point-of-care wound diagnostics (Figure 2).

## 4. Panel Recommendations

### 4.1. Question 1: Which Test Should I Use First, BPA or EPA?

**Figure 3:**
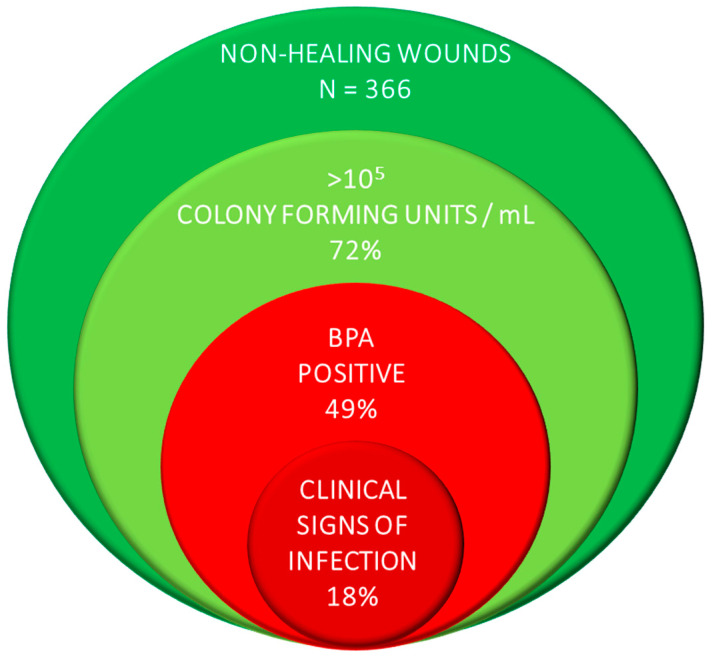
A large multicenter clinical trial demonstrates that wounds with pathogenic bacteria have no signs or symptoms of infection.

The consensus panel concluded that clinicians should test for BPA first. The panel maintained that the risk of invasive infection resulting from the presence of pathogenic bacteria was the greatest threat to the patient’s wellbeing. It was also recommended that clinicians consider testing patients with wounds that have failed to progress to rule out pathogenic bacteria as the cause for delayed wound healing. The opinion is supported by evidence from a multicenter clinical trial in which 39% of wounds had elevated BPA levels in the absence of signs of infection (Figure 3) [19].

If the BPA test is negative, the panel recommended testing for EPA. In addition, it was suggested that if the wound failed to progress after the elevated BPA was treated and subsequent testing was negative for BPA, the clinician should consider testing for EPA.

### 4.2. Question 2: What Is the Best Therapy Pathway Once I Get the Test Results?

The consensus panel recommended that the treatment for wounds testing BPA positive without three or more clinical signs of infection consist of wound cleansing, debridement, and application of a topical antimicrobial. If clinical signs and symptoms of infection are present, consider culturing the wound (swab, biopsy or molecular diagnostics) and adding systemic antibiotic therapy. If a chronic wound has a negative BPA test, consider inflammation as a cause for delayed wound healing and test for EPA.

For an EPA result, the panel suggests cleansing the wound with an antiseptic, debridement, and protease modulation (e.g., collagen dressings, oral doxycycline, ORC/collagen).

### 4.3. Question 3: When Should I Retest?

The panel agreed that wounds testing positive for BPA should be retested in two weeks. If after two weeks the test result remains positive, they recommended cleansing the wound with an antiseptic, debriding the wound, applying a topical antimicrobial, and considering adding oral antibiotic therapy either empirically or based on culture results. Retesting again in 10 days to two weeks is also suggested. If the wound remains positive, reassess the wound, obtain a culture (swab, biopsy, or molecular diagnostic), adjust antibiotic therapy, cleanse the wound with an antiseptic, and perform debridement.

Similarly, if the wound tests positive for EPA retest in two weeks. If after two weeks EPA persists, continue cleansing with an antiseptic, debride the wound, and adjust inflammatory modulation therapy. Retest again in two weeks. If the four week test result is still positive, consider “resetting” the wound. This involves extensive debridement and oral doxycycline in combination with additional protease modulation therapy.

## 5. Summary of Therapy Pathway the Table Outlines a Plan of Care Based on the Results of the BPA and EPA Results



**EPA Elevated**

**EPA Not Evevated**
BPA Elevated ■Debride the wound■Cleanse the wound with an antiseptic■Treat with a broad-spectrum topical antimicrobial■Retest in two weeks■If BPA remains positive, continue antiseptic, culture the wound, apply an antimicrobial dressing and consider a culture-directed topical antibiotic■Retest in two weeks■If BPA is still positive, perform aggressive sharp debridement, apply a topical antimicrobial, reassess, or start an oral antibiotic
Note: If at any time the BPA becomes negative and the wound is not improving, retest for EPA.
■Focus on resolving the infection
BPA Not Elevated
■Cleanse the wound with antiseptic or saline■Debridement as indicated■Treat with inflammatory protease modulation therapy■Retest in two weeks■If EPA persists, consider doxycycline in combination with topical protease modulation therapy■Retest every two weeks until proteases are negative.■Do not proceed with an advance modality therapy (e.g., grafts)

■Standard of care


## 6. Additional Considerations

### 6.1. Wound Bed Preparation Prior to Advanced Therapies

The panel strongly recommended the application of advanced products (e.g., cellular or tissue-based products) only when BPA is negative and EPA is low.

### 6.2. Antimicrobial Stewardship

Starting in 2020, Joint Commission requires all outpatient departments in the United States that prescribe antibiotics to develop an antibiotic stewardship program [20]. Incorporating point-of-care testing, such as BPA, into an antimicrobial stewardship may increase the detection of pathogenic bacteria in the absence of CSS allowing for more appropriate use of topical antimicrobials and system antibiotics.

## 7. Conclusions

The panel expects that following the pathway outlined in this manuscript will improve healing outcomes, result in more cost-effective use of advanced therapies, and improve antimicrobial stewardship by guiding antimicrobial use.
